# Antitumor Effects of a New Retinoate of the Fungal Cytotoxin Illudin M in Brain Tumor Models

**DOI:** 10.3390/ijms23169056

**Published:** 2022-08-13

**Authors:** Benedikt Linder, Miroslava Zoldakova, Zsuzsanna Kornyei, Leonhard H. F. Köhler, Sebastian Seibt, Dominic Menger, André Wetzel, Emília Madarász, Rainer Schobert, Donat Kögel, Bernhard Biersack

**Affiliations:** 1 Experimental Neurosurgery, Frankfurt University Hospital, Theodor-Stern-Kai 7, 60590 Frankfurt am Main, Germany; 2 Organic Chemistry 1, University of Bayreuth, Universitätsstrasse 30, 95440 Bayreuth, Germany; 3 Laboratory of Cellular and Developmental Neurobiology, Institute of Experimental Medicine of the Hungarian Academy of Sciences, Szigony utca 43, HU-1083 Budapest, Hungary

**Keywords:** illudin M, retinoic acid, neuronal cells, brain tumors, anticancer agents

## Abstract

While the fungal metabolite illudin M (**1**) is indiscriminately cytotoxic in cancer and non-malignant cells, its retinoate **2** showed a greater selectivity for the former, especially in a cerebral context. Illudin M killed malignant glioma cells as well as primary neurons and astrocytes at similarly low concentrations and destroyed their microtubule and glial fibrillary acidic protein (GFAP) networks. In contrast, the ester **2** was distinctly more cytotoxic in highly dedifferentiated U87 glioma cells than in neurons, which were even stimulated to enhanced growth. This was also observed in co-cultures of neurons with U87 cells where conjugate **2** eventually killed them by induction of differentiation based on the activation of nuclear receptors, which bind to retinoid-responsive elements (RARE). Hence, illudin M retinoate **2** appears to be a promising drug candidate.

## 1. Introduction

The sesquiterpene illudin M (**1**) was first isolated from the culture broth of *Omphalotus olearius* mushrooms [1]. After a pre-activating reduction of its enone by NADPH-dependent oxido-reductases or glutathione (Nu^1^), **1** can alkylate DNA, RNA and proteins (Nu^2^) via opening of the spirocyclopropane and thereby induce apoptotic cell death [2]. Although illudin M, similar to its congener illudin S and the related semisynthetic irofulven, is highly efficacious against cancer cells, its indiscriminate toxicity has prevented clinical applications [3,4]. Occasionally, illudin derivatives with reduced toxicity and improved therapeutic indices were reported [5]. Irofulven even underwent several phase II clinical trials but proved largely ineffective, except for some prostate and pancreatic cancers [6].

Retinoic acid (RA) induces differentiation of various types of stem cells, including cancer and neural stem cells, and a retardation of cancer cell proliferation [7,8]. These effects are mediated by nuclear retinoic acid receptors, usually consisting of heterodimers between RAR and RXR proteins, and sometimes by interfering with estrogen signaling [9,10]. Retinoids were particularly efficacious against various human carcinomas when applied as part of combination regimens with standard drugs such as cisplatin or with HDAC and DNA methyltransferase inhibitors [11,12]. Here, we report on the selective impact of a new illudin M retinoate **2** on glioma and stem-cell rich astrocytoma cells, when compared with normal neurons and astrocytes.

## 2. Results

**Scheme 1:**
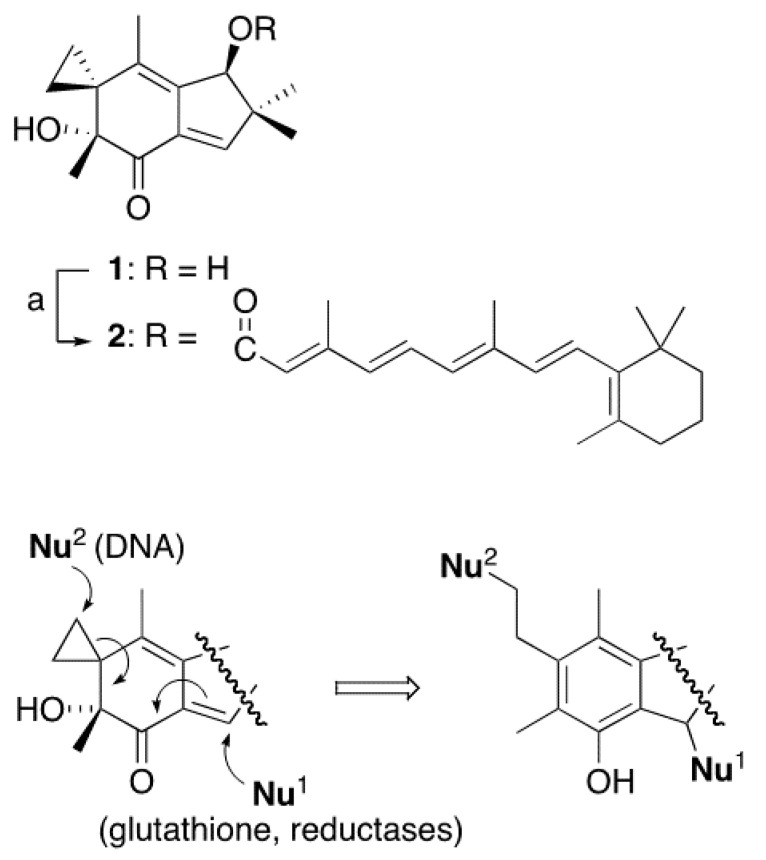
Synthesis of the retinoate **2** of illudin M (**1**) and mechanism of action. Reagents and conditions: (a) retinoic acid, C_6_H_2_Cl_3_COCl, DMF, NEt_3_, then **1**, DMAP, toluene, 16 h, r.t., 41%.

Retinoate **2** was prepared by esterification of all-*trans* retinoic acid with **1** under Yamaguchi conditions (Scheme 1) [13]. The antiproliferative activities of compounds **1** and **2** were first evaluated by cell viability (MTT) assays against a panel of seven cancer and three non-malignant cell types. These comprised human 518A2 melanoma, HL-60 leukemia, the multi-drug resistant carcinomas KBv1^+Vbl^ cervix and MCF-7^+Topo^ breast, the HT-29 colon carcinoma, the cancer stem cell-rich rat C6 astrocytoma, human U87 glioma as well as non-malignant primary mouse astrocytes and neurons, and the NE-4C neuroectodermal stem cells isolated from the fore- and midbrain vesicles of p53-deficient 9-day-old mouse embryos [14]. Generally, the retinoate **2** was 100-fold less efficacious than **1** against the non-neural cancer cell lines, with the exception of MCF-7^+Topo^, which responded equally and well to both compounds (Table 1). This cell line is estrogen-dependent and also overexpresses ABC-transporters of the BCRP (breast cancer resistance protein) type, which are assumed to contribute to cancer stem cell resistance and inefficient trespassing of some drugs through the blood brain barrier [15,16]. Against the brain-derived cells, e.g., neuronal progenitors (NE-4C), neurons, astrocytes (Table 2), and glioma lines C6, U87, U251, and MZ-54 (Table 1), **1** again showed similar activities with IC_50_ (48 h) values ranging from 50 to 400 nM. In contrast, the retinoate **2**, while being generally less active than **1**, displayed a significantly greater cytotoxicity against the human U87, U251 and MZ-54 glioblastoma cells than against normal neurons and astrocytes (Table 1 and Table 2). The NE-4C neuronal progenitor cells were also sensitive to treatment with compound **2** (Table 1).

**Figure 1:**
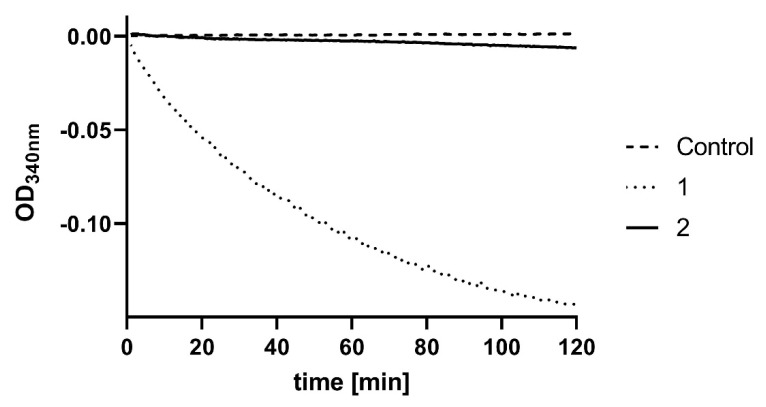
Time-resolved measurement of the absorption at 340 nm (enone group) of 200 µM **1**, **2** or solvent (DMSO) mixed with 6.5 mM glutathione in phosphate-buffered saline (PBS). Data are means ± SD of at least four independent measurements every 30 s.

Compound **1** is a natural prodrug and undergoes activation by reaction with bionucleophiles such as glutathione forming an activated cyclopropane, which readily reacts with other bionucleophiles, for example, nucleobases of DNA (Scheme 1). The reactivity of the illudin M scaffold was significantly reduced by esterification, explaining the higher antiproliferative activity and toxicity of **1** when compared with ester conjugates [13]. In order to investigate whether retinoic conjugate **2** is also less reactive than **1**, the fading of the enone band of compounds **1** and **2** was monitored at 330 nm in the presence of glutathione, showing a stabilization of conjugate **2** compared with **1** (Figure 1). The stability of conjugate **2** in cell medium was also investigated using HPLC techniques, and **2** showed a high stability under these conditions (cf. Table S1, Supporting Information). A cleavage of the ester bond over the time of observation can be ruled out as neither free **1** nor free all-*trans* retinoic acid were detectable by HPLC. Thus, the anticancer effects of **2** are not based on any **1** formed upon degradation of **2**, but on the intact molecule.

**Figure 2:**
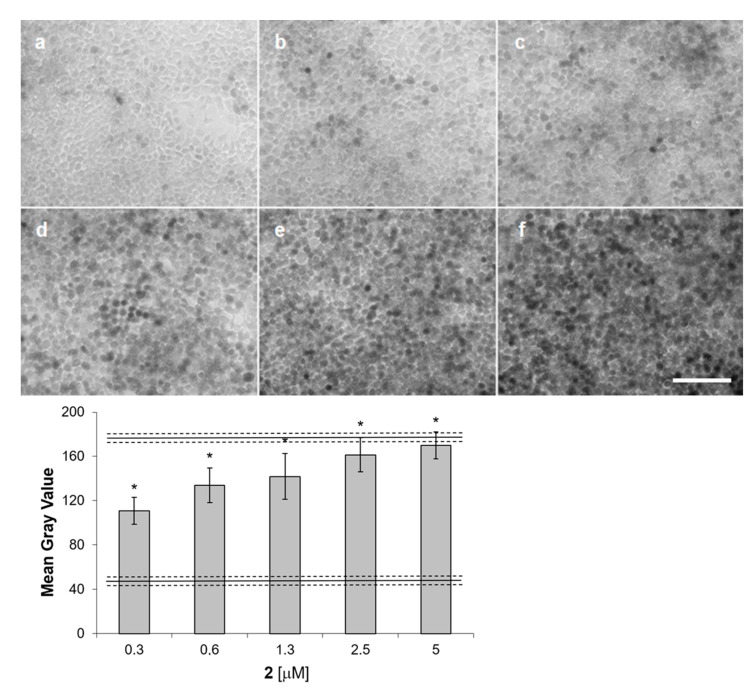
F9 RARE-*lacZ* reporter cells show concentration-dependent activation upon treatment with **2** for 20 h. Top: bright-field images of cells treated with **2** ((**a**): 0 µM, (**b**): 0.3 µM, (**c**): 0.6 µM, (**d**): 1.3 µM, (**e**): 2.5 µM, (**f**): 5.0 µM), and β-galactosidase activity was visualized by X-Gal staining (scale bar: 100 µm). Bottom: densitometric image analyses (mean ± SD); a greater gray value means stronger RARE binding. The threshold lines at 45 and 175 represent the control and the effect of 10 nM all-*trans* retinoic acid. * *p* < 0.001, Student’s t test, groups treated with **2** were compared to control, *n* = 5.

More typical of retinoic acid is its contribution to cell differentiation during vertebrate embryonic development by interacting with nuclear receptors, which bind to specific retinoid-responsive elements (RARE) within the promoters of subordinate genes, activating their transcription [17,18,19,20]. To verify that retinoate **2** also binds to RARE, we used a reporter assay with F9 teratocarcinoma cells featuring a RARE located within the *cis*-acting regulatory sequences of the human retinoic acid receptor alpha gene and immediately upstream of the *E. coli lacZ* genes, which thus acquire retinoid responsivity [21]. *LacZ* expression in cells treated with various concentrations of **2** was then quantitated by densitometric analysis of β-galactosidase activity visualized by X-Gal staining [22]. Figure 2 shows that compound **2** bound to the F9 RARE-*lacZ* reporter cells in a concentration-dependent manner.

**Figure 3:**
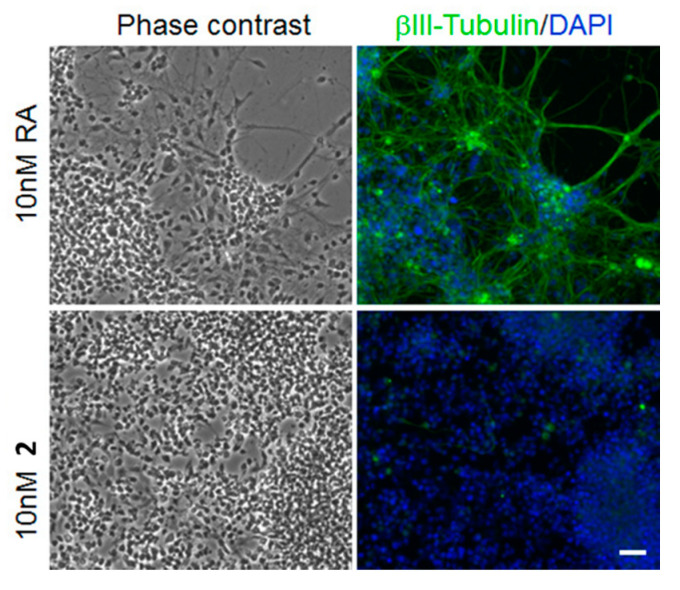
Upper row: NE-4C neural stem cells differentiate to neurons upon treatment with 10 nM of all-*trans* retinoic acid (RA). Bottom row: retinoate **2** (10 nM) did not elicit differentiation of NE-4C stem cells. Right column shows immunohistographs after staining for neuron specific ß-tubulin III (green) and for nuclei with DAPI (blue). Scale bar: 50 µm.

**Figure 4:**
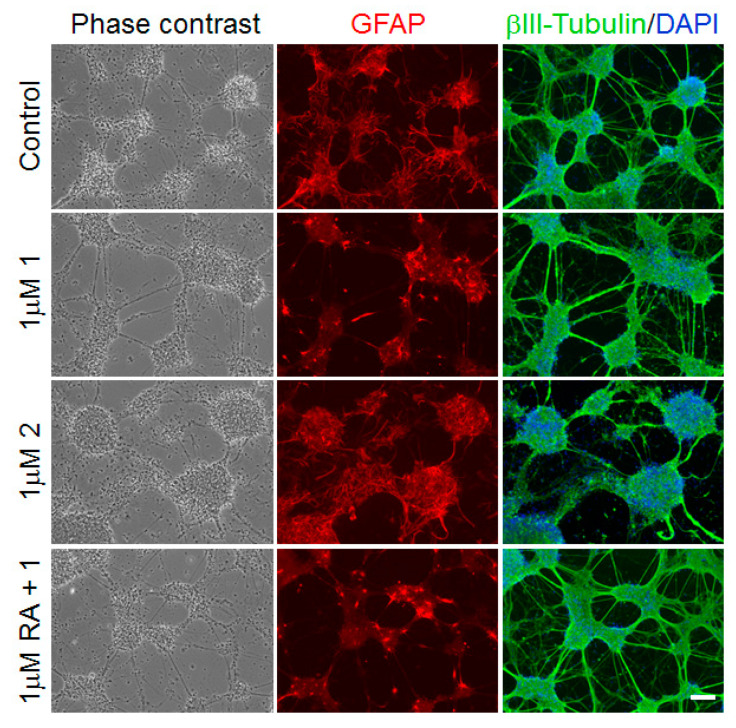
Immunostaining of primary neurons derived from E14, 5 forebrains treated with 1 µM of **1**, **2** or all-*trans* retinoic acid (RA) plus **1**. Blue: nuclei stained with DAPI; green: tubulin stained with βIII-tubulin specific antibody; red: GFAP stained with α-GFAP antibody. Fluorescence microscope Zeiss Axiovert 200 M, 63× objective, 1024 × 1024 res. Scale bar: 50 µm.

**Figure 5:**
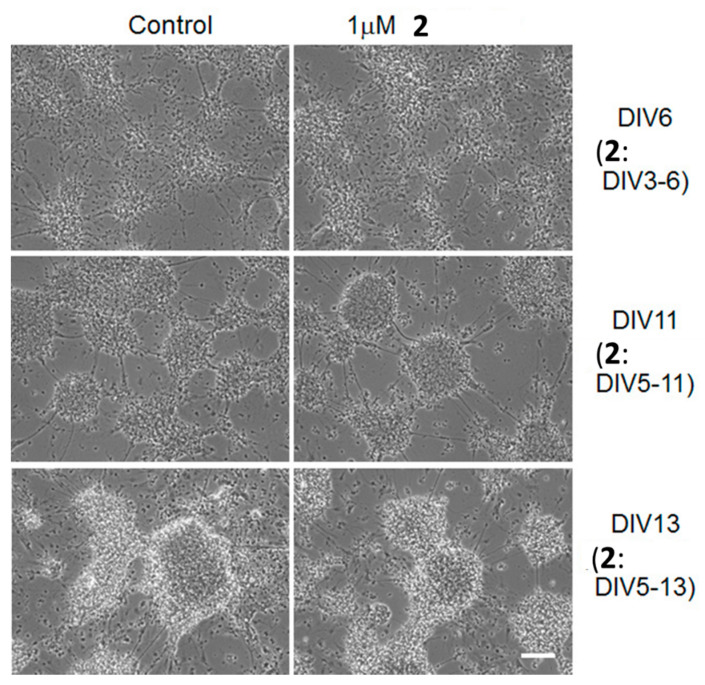
Micrographs of cultures of mouse embryonal neuronal cells untreated (control) or treated with 1 µM of **2**; DIV, days in vitro. Scale bar: 100 µm.

While binding to the RARE of these reporter cells, retinoate **2**, unlike all-*trans* retinoic acid itself, did not induce neuronal differentiation of NE-4C neural stem cells even at concentrations as high as 1 µM. NE-4C cells and their differentiated progeny neurons can be immunohistochemically distinguished by the presence of β-tubulin III only in the latter (Figure 3). While short term (48 h) **1** application (1 µM) did not completely disorganize neuronal networks, it affected astrocytes within neuronal cultures, resulting in altered expression/localization of glial fibrillary acidic protein (GFAP), a major cytoskeletal element of glial cells responsible for maintaining mechanical cell stability [23]. As illustrated by fluorescent immunocytochemistry, GFAP filaments were markedly pitted after 48 h exposure to **1**, while retinoate **2** did not cause similar alterations to astrocytes (Figure 4). Moreover, neuronal cells developed normally when exposed to 1 µM retinoate **2** for as long as 3, 6 or 8 days (Figure 5).

**Figure 6:**
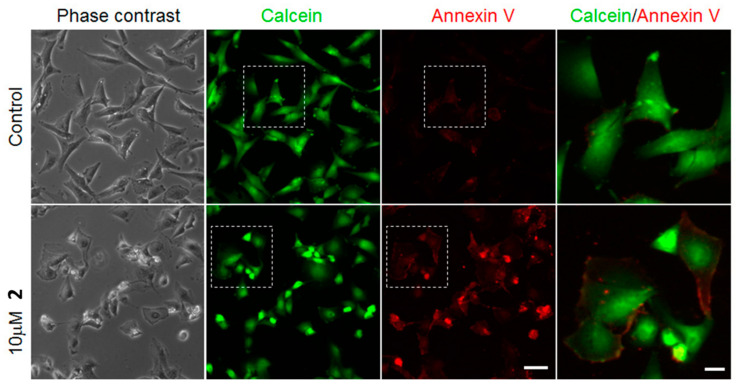
U87 glioma cells undergo apoptosis upon 48 h treatment with 10 µM of **2**. Cells in the early stages of apoptosis show calcein (green)/Annexin V-Cy3 (red) double labeling. Scale bars: 50 µm; 20 µm for inserts.

**Figure 7:**
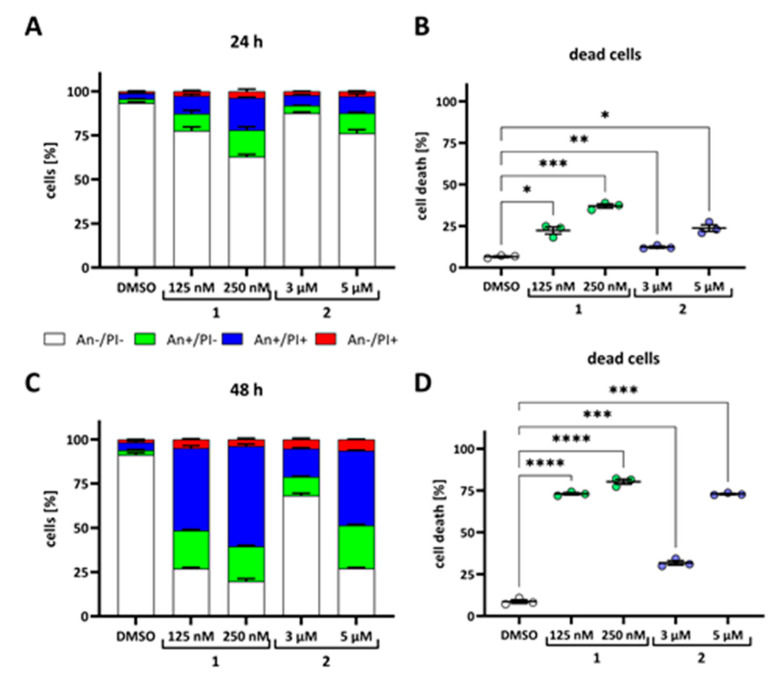
(**A**–**D**)**.** Annexin V/PI flow cytometry of MZ–54 cells upon treatment with **1** or **2**, mean + SEM; Brown–Forsythe ANOVA test with Dunnett’s T3 multiple comparison test (compared with DMSO control); * < 0.05; ** < 0.01; *** < 0.001; **** < 0.0001.

In contrast to astrocytes and neuronal cells, cultures of U87 glioma cells were far more sensitive to retinoate **2** at concentrations between 1 and 10 µM, undergoing rapid apoptosis (Figure 6). Further studies on the pro-apoptotic effects of **1** and **2** were carried out with MZ-54 glioblastoma cells. Annexin V/propidium iodide (PI) flow cytometry of MZ-54 cells treated with **1** or **2** revealed a dose- and time-dependent increase in cell death (Figure 7).

**Figure 8:**
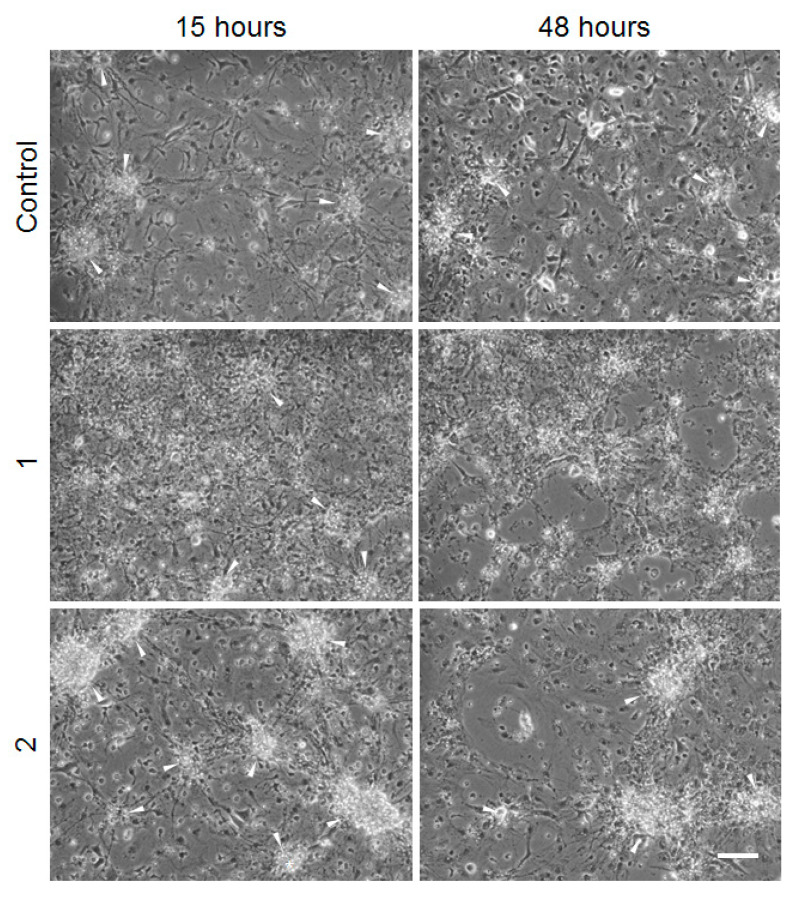
Co-culture of neuronal cells and U87 glioma cells in the absence or presence of 1 µM of **1** or **2**. The neuronal aggregates are marked by arrowheads. Scale bar: 100 µm.

The selectivity of illudins **1** and **2** for U87 glioblastoma cells versus normal neuronal cells was evaluated by applying them to co-cultures of these cells serving as a surrogate brain tumor model. To this end, isolated murine primary neurons were grown on IBIDI dishes and after 5 days and the establishment of neuronal aggregates, the glioma U87 cells (~ 25,000 cells) were added to the neuronal culture [24]. After 12 h, **1** and **2** were applied in 1 µM concentrations and the response of the different cells was monitored over a period between 15 and 48 h post incubation (Figure 8). In the control samples, the number of proliferating U87 cells increased with time and overgrew the diminishing number of differentiating neurons. In contrast, in the samples treated with **1**, both the neurons and the U87 cells had undergone considerable cellular fragmentation as early as 15 h after treatment, leaving merely cell debris after 48 h. Co-cultures incubated with retinoate **2** featured healthy and compact neuronal aggregates (not quantified numerically) after 15 h compared with control cells. After 48 h exposure to **2**, many neurons were still present in the culture.

**Figure 9:**
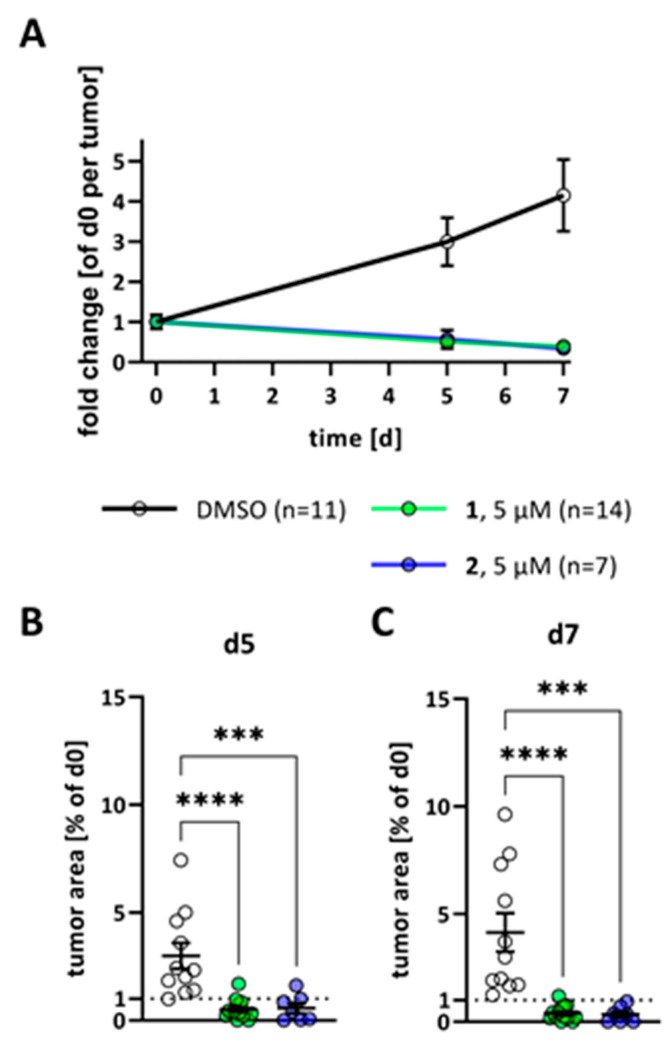
Ex vivo tumor growth assay using organotypic brain slice cultures in the absence (DMSO) or presence of 5 µM of **1** or **2**. (**A**) Growth curves of the NCH644^GFP+^ tumors over time after treatment with solvent (DMSO, black line) or 5 µM **1** (green line) or **2** (blue line) normalized to the tumor size one day after transplantation (d0) depicted as mean +/− SEM. (**B**,**C**) Point plots of the data summarized in (**A**) after treatment for (**B**) 5 days or (**C**) 7 days). Dashed line on y = 1 display the original tumor size. ***: *p* < 0.001; ****: *p* < 0.0001; two-Way ANOVA with Tukey’s multiple comparisons test (GraphPad Prism 7).

**Figure 10:**
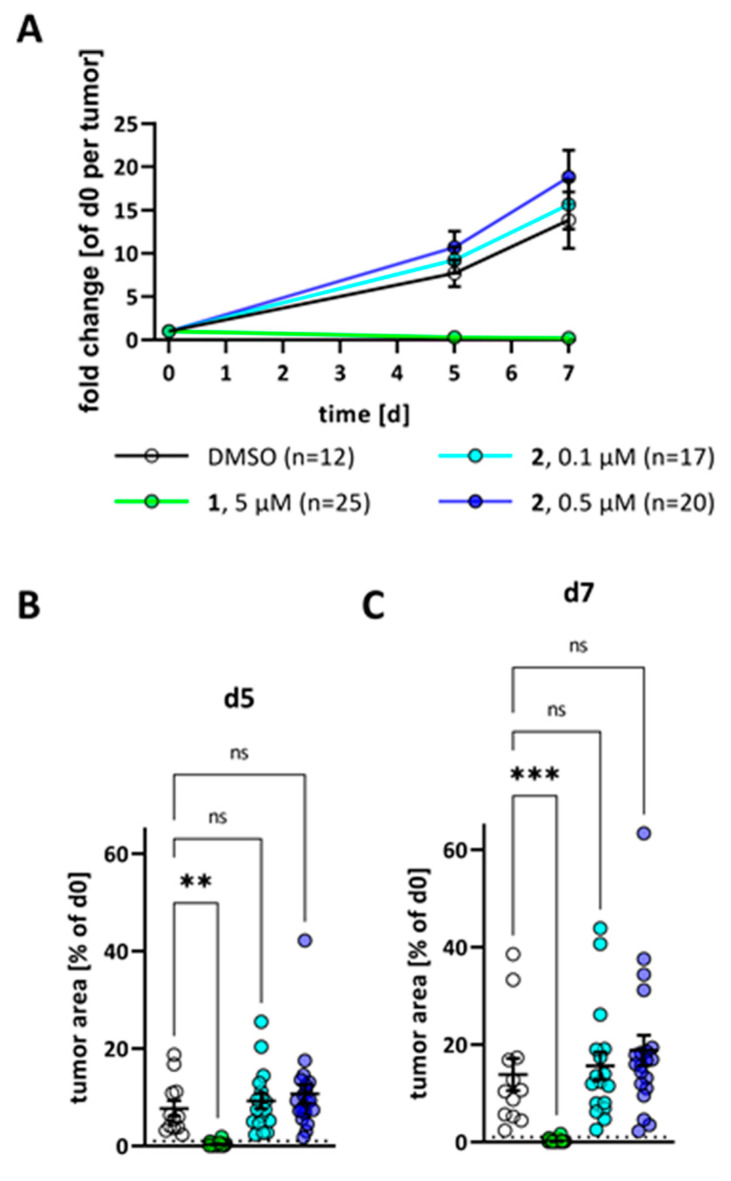
Ex vivo tumor growth assay using organotypic brain slice cultures in the absence (DMSO) or presence of 5 µM of **1** or 0.1 µM or 0.5 µM **2**. (**A**) Growth curves of the NCH644^GFP+^ tumors over time after treatment with solvent (DMSO, black line) or 5 µM **1** (green line), 0.1 µM (light blue line) or 0.5 µM **2** (blue line) normalized to the tumor size one day after transplantation (d0) depicted as mean +/− SEM. (**B**,**C**) Point plots of the data summarized in (**A**) after treatment for (**B**) 5 days or (**C**) 7 days). Dashed line on y = 1 display the original tumor size. **: *p* < 0.001; ***: *p* < 0.0001; two-way ANOVA with Tukey’s multiple comparisons test (GraphPad Prism 7).

For further investigation of drug efficacy and selectivity, an **ex vivo** tumor growth assay employing organotypic brain slice cultures of adult murine brains were used. For this purpose, GFP-expressing NCH644 (NCH644^GFP+^) glioma stem-like cells were transplanted onto these slices, and after 1 day, the treatment was started and renewed three times per week, similar to our previous studies [25,26]. These cultures are suitable ex vivo models, which simulate original brain architecture and the presence of vessels, including angiogenesis and microvascular proliferation as well as specific homing of GBM cells toward those vessels, while requiring much fewer laboratory animals when compared with common in vivo animal drug tests [27,28,29]. Both **1** and **2** showed antitumor activity in the organotypic brain cancer model using NCH644^GFP+^ at a dose of 5 µM (Figure 9). The activity of **2** was significantly reduced at doses of 0.1 and 0.5 µM, which is in line with results from the in vitro MTT experiments, hinting at a considerable stability of the ester conjugate **2** under these conditions (Figure 10).

## 3. Discussion

Illudin M (**1**) is a fungal product with a unique DNA-damaging mechanism. However, its high toxicity toward laboratory animals stopped its development as an anticancer agent at an early stage [5]. Esterification of the secondary alcohol of **1** led to compounds with reduced toxicity and increased selectivity [13]. Conjugation of **1** to *all*-trans retinoic acid via ester bonding led to remarkable results. In vitro MTT experiments revealed that MCF-7^+Topo^ breast carcinoma cells responded well to ester **2**. These cells are multidrug-resistant cells generated by the exposure to topotecan. In addition to their hormone-sensitive character, they overexpress the BCRP (breast cancer resistance protein) transporter, which plays a role for cancer stem cell resistance, and impaired crossing of drugs through the blood brain barrier [15,16]. The strong time dependence of the activity of ester **2** in the MCF-7^+Topo^ breast carcinoma cells can be a hint at a metabolism of **2** in these cells leading to active illudin M. Similarly, a strong time dependence of compound **2** activity was observed in HT-29 colorectal cancer cells, which are also ABC-transporter expressing multidrug-resistant cells [30]. The multidrug-resistant phenotype is characterized by an increased pH value and alkalinity in the cytoplasm of cancer cells, which might facilitate the hydrolysis of retinoate **2** and the release of **1** from **2** in these cells [31,32]. In addition, U87 glioblastoma cells exhibited strong time-dependent sensitivity to compound **2**. Although intrinsically not multidrug-resistant, ABCG2/BCRP expression can be induced in U87 cells by treatment with anticancer active drugs such as perphenazine and prochlorperazine [33]. If this is also the case for **2**, it remains to be elucidated.

Ester **2** unlike **1** is distinctly more cytotoxic in the cancer stem cell-rich rat C6 astrocytoma cell line than in normal astrocytes. This is somewhat paradoxical since retinoic acid is a known inhibitor of c-Jun N-terminal kinase (JNK), and illudin derivatives such as irofulven were shown to induce apoptosis via activation of JNK and ERK [34,35,36]. However, C6 astrocytoma cells, unlike normal astrocytes, naturally express cannabinoid CB_1_ receptors, which enhance JNK activity upon binding of certain unsaturated fatty acids such as arachidonates, and maybe also of retinoic acid [37,38]. The latter was found to induce apoptosis in C6 glioma cells at relatively high concentrations [39].

The new illudin M retinoate **2** exhibited distinct antiproliferative and apoptosis-inducing effects on various glioblastoma cells. The high selectivity of **2** was confirmed in the co-culture assay using U87 glioma cells and neuronal cells and is corroborated by the high stability of this ester conjugate in cell medium under the conditions applied for the in vitro experiments. Finally, our application of the compound in an organotypic model further underscored the selective targeting of tumor cells within a complex non-transformed environment, since no signs of toxicity could be observed in the tumor-free regions of the brain slices. OTCs are a widely used, 3R-compatible and valuable ex vivo method to recapitulate the physiological brain environment of GBM over an extended time period. This demonstrates vividly the selective antitumor activity of compound **2**. In addition, compound **2** was able to exert its effects via RARE in F9 embryonal carcinoma cells, while it kept its illudin-type properties to a certain extent. This observation indicates an excellent interplay of the retinoate and illudin moieties of the conjugate molecule **2** and warrants a more detailed study of its mechanism of action in tumor cells vs. healthy cells, including models allowing to assess/compare drug effects in a more authentic brain environment. Further studies are planned in order to substantiate the drug-like properties of **2** and the potential of **2** as a promising drug candidate against glioma and multidrug-resistant tumors. This approach will also uncover if **2** can effectively cross the blood–brain barrier (BBB). Other illudin derivatives such as irofulven have been shown to enhance survival of intracranial glioma xenografts in mice, suggesting active crossing of the BBB [40].

## 4. Materials and Methods

### 4.1. General

Starting materials and reagents were purchased from Sigma-Aldrich (Taufkirchen, Germany). Illudin M was isolated according to published procedures [13]. The following instruments were used: melting points (uncorrected), Gallenkamp (Cambridge, UK); IR spectra, Perkin-Elmer Spectrum One FT-IR spectrophotometer (Rodgau, Germany) with ATR sampling unit; nuclear magnetic resonance spectra, BRUKER Avance 300 spectrometer (Billerica, MA, USA); chemical shifts are given in parts per million (δ) downfield from tetramethylsilane as internal standard; mass spectra, Varian MAT 311A (EI, Palo Alto, CA, USA).

### 4.2. Synthesis of Compound ***2***

Retinoic acid (100 mg, 0.33 mmol) was suspended in dry DMF (1 mL), and Et_3_N (53 µL, 0.36 mmol) and 2,4,6-trichlorobenzoyl chloride (59 µL, 0.36 mmol) were added. The reaction mixture was stirred at room temperature for 20 min. Illudin M (83 mg, 0.33 mmol) and DMAP (81 mg, 0.66 mmol) dissolved in dry toluene (5 mL) were added and the reaction mixture was stirred at room temperature for 16 h. After dilution with ethyl acetate and washing with water, the organic phase was dried over Na_2_SO_4_ and concentrated in vacuum. The residue was purified by column chromatography (silica gel 60). Yield: 64 mg (0.12 mmol, 36%); yellow oil; *R*_f_ = 0.61 (ethyl acetate/*n*-hexane 1:4); *ν*_max_ (ATR)/cm^−1^: 3492, 2962, 2928, 2866, 1696, 1606, 1580, 1447, 1360, 1255, 1235, 1139, 1105, 966, 945, 821, 730; ^1^H-NMR (300 MHz, CDCl_3_): δ 0.3–0.5 (1 H, m, 9-H^a^), 0.8–1.0 (2 H, m, 8-H^a^, 9-H^b^), 1.00 (6 H, s, 2 × Me), 1.1–1.6 (17 H, m, 4 × Me, 2 × CH_2_, 8-H^b^), 1.69 (3 H, s, Me), 1.9–2.1 (5 H, m, Me, CH_2_), 2.35 (3 H, s, Me), 3.55 (1 H, s, OH), 5.68 (1 H, s, 3-H), 5.77 (1 H, s, 2′-H), 6.0–6.3 (4 H, m, 4′-H, 6′-H, 8′-H, 9′-H), 6.50 (1 H, s, 1-H), 6.9–7.1 (1 H, m, 5′-H); ^13^C-NMR (75.5 MHz, CDCl_3_): δ 6.0 (C-8), 8.8 (C-9), 12.9 (7′-Me), 14.0 (3′-Me), 14.5 (4-Me), 19.2 (cyclohexyl-CH_2_), 20.8 (2-Me), 21.7 (cyclohexyl-C*Me*), 24.8 (6-Me), 26.8 (2-Me), 29.0 (cyclohexyl-C*Me_2_*), 31.5 (C-5), 33.1 (cyclohexyl-CH_2_), 34.3 (cyclohexyl-*C*Me_2_), 39.6 (cyclohexyl-CH_2_), 49.0 (C-2), 76.1 (C-6), 78.1 (C-3), 117.8 (C-2′), 128.9 (C-9′), 129.4 (C-6′), 130.1 (cyclohexyl-*C*Me), 131.4 (C-5′), 133.6 (C-7a), 134.9, 135.1 (C-3a, C-4), 137.2 (C-4′), 137.7 (cyclohexyl-*C*=CMe), 140.0 (C-7′), 146.5 (C-1), 153.7 (C-3′), 166.9 (CO_2_), 200.3 (CO); *m/z* (EI) 293 (13), 282 (13), 231 (11), 209 (20), 177 (21), 84 (100).

### 4.3. Cells and Cell Culture

518A2 (Department of Radiotherapy, Medical University of Vienna, Vienna, Austria) melanoma, KB-V1^Vbl^ (ACC-149) cervix carcinoma, MCF-7^Topo^ (ACC-115) breast carcinoma, HT29 (ACC-299) colon carcinoma were cultivated in Dulbecco’s Modified Eagle Medium (DMEM) supplemented with 10% fetal bovine serum and 1% antibiotic-antimycotic at 37 °C, 5% CO_2_, and 95% humidity. To keep MCF-7^Topo^ and KB-V1^Vbl^ cells resistant, the maximum-tolerated doses of topotecan or vinblastine were added to the cell culture medium 24 h after every passage. The human HL60 leukemia cells were obtained from the German National Resource Center for Biological Material (DSMZ), Braunschweig. Neuroectodermal stem cells NE-4C (ATCC No. CRL-2925), primary mouse astrocytes, rat glioma C6 (ATCC No. CCL-107) and human glioma U87 (ATCC No. HTB-14) were used [41].

NE-4C cells derived from the anterior brain vesicles of p53-deficient 9-day-old mouse embryos were maintained in Minimum Essential Medium (MEM; Sigma Aldrich, Taufkirchen, Germany) supplemented with 5% fetal calf serum (FCS; Invitrogen-Gibco, Carlsbad, CA, USA), 4 mM glutamine (Sigma-Aldrich, Taufkirchen, Germany) and 40 ug/mL gentamycin (Chinoin, Budapest, Hungary). For maintenance, subconfluent cultures were regularly split by trypsinization (0.05 *w*/*v*% trypsin in PBS) into poly-L-lysine-coated Petri dishes. Primary astrocytes were isolated from whole brains of neonatal (P0-P3) mice, as described earlier [24]. In brief, meninges were removed, and the tissue pieces were subjected to enzymatic dissociation, using 0.05% *w*/*v* trypsin and 0.05% *w*/*v* DNase for 10 min at room temperature. The cells were plated onto poly-L-lysine coated plastic surfaces and grown in MEM supplemented with 10% fetal calf serum, 4 mM glutamine and 40 ug/mL gentamycin. Primary neuronal cultures were prepared from embryonic (E15–E16) mouse (CD1) forebrains, as described earlier [42]. In brief, cell suspensions were prepared by mechanical dissociation over a nylon mesh with a pore diameter of 40–42 μm. The cells were seeded at 10^5^ cells/cm^2^ density onto PLL-coated surfaces. The cultures were maintained in MEM supplemented with 5% FCS, 4 mM glutamine, 40 µg/mL gentamicin and 2.5 μg/mL amphotericin B (Sigma). In co-culture experiments, U87 glioma cells were seeded onto established neuronal cultures at DIV3 in a 25,000 cell/cm^2^ density.

U251 (formerly known as U-373 MG; ECACC 09063001), Mz54 (CVCL_M406) human glioblastoma cell lines were cultured in DMEM+GlutaMax supplemented with 10% fetal bovine serum and 1% penicillin/streptomycin mixture. The human glioma cell line Mz54 was obtained from the Dept. of Neurosurgery, University Medical Centre, Johannes Gutenberg University Mainz, Germany, where this line was isolated from a recurrent grade IV glioblastoma. U251 and Mz54 cells were used in passages between 10 and 25 after re-authentication and 40 to 55 after culture establishment, respectively. Only mycoplasma-free cell cultures were used.

NCH644^GFP+^ have been described previously and are derived from NCH644, which were kindly provided by Christel Herold-Mende (University Hospital Heidelberg, Germany) and were cultured as free-floating spheres in serum-free medium [34,35,43]. Specifically, the cells were cultured in Neurobasal-A medium (Gibco, Darmstadt, Germany) supplemented with 1× B27 (Gibco), 100 U/mL penicillin, 100 µg/mL streptomycin (P/S, Gibco), 1 × GlutaMAX (Gibco), 20 ng/mL epidermal growth factor (EGF, Peprotech, Hamburg, Germany), 20 ng/mL fibroblast growth factor (FGF, Peprotech) and 2 µg/mL puromycin for GFP-expressing cells.

### 4.4. Cell-Based Assay

Non-GBM cells (5 × 10^4^ cells/mL, 100 µL/well) were grown in 96-well plates for 24 h. Then, they were treated with various concentrations of the test compounds or vehicle (DMSO, or EtOH) for 72 h at 37 °C. After the addition of 12.5 µL of a 0.5% MTT solution in PBS, the cells were incubated for 3 h at 37 °C so that the water-soluble MTT could be converted to formazan crystals. Then, the plates were centrifuged (300× *g*, 5 min, 4 °C), the medium withdrawn, and the formazan dissolved in 25 µL of DMSO containing 10% sodium dodecylsulfate (SDS) and 0.6% acetic acid for at least 2 h at 37 °C. In the case of the HL60 cells, after 2 h, the precipitate of formazan crystals was redissolved in a 10% solution of SDS in DMSO containing 0.6% acetic acid.

Adherently grown GBM cells were seeded at 5 × 10^4^ cells/mL and floating spheres at 8 × 10^4^ cells/mL in 100 µL/well and incubated as above for 48 or 72 h at 37 °C. Afterward, 10 µL of a 5 mg/mL MTT solution in PBS was added to the cells for 3 h at 37 °C. Then, the spheres were centrifuged shortly to collect the cells on the bottom of the plate, and the medium was removed by careful pipetting for both adherently grown and floating sphere GBM cultures. Afterward, formazan was dissolved in a mixture (24:1 *v*:*v*) of isopropanol and 1 M HCl using 100 µL for adherent cells by shaking the plates for at least 20 min. The absorbance of formazan (λ = 570 nm), and background (λ = 630 nm) was measured with a microplate reader (Tecan Spark, Tecan Deutschland GmbH, Crailsheim, Germany).

### 4.5. RA Reporter Assay

The F9 embryonal carcinoma cell line, stably transfected with the 1.8 kb promoter sequence of RARb2 coupled to the *lacZ* gene, was used to measure active retinoids [44]. The assay is appropriate for detection of all-*trans* RA but is able to detect other retinoid isomers as well. The F9 reporter cells were maintained in 10% FCS containing DMEM in the presence of 400 ug/mL G418. A day before the assay, F9 cell were seeded onto 24-well plates (100,000 cell/well). The cells were than treated with various concentrations of **2**. After 20 h, the β-galactosidase activity was visualized by X-Gal staining, which was followed by densitometric image analyses from *n* = 3 fields of view (10× magnification) of *n* = 4 independent cultures.

### 4.6. Reactivity Test with Glutathione

In a cell-free activity assay, the reaction of **1** and **2** with glutathione was measured by the decreasing absorption at 340 nm (enone group). Then, 2 µL of the substances (10 mM in DMSO) was mixed with 98 µL glutathione solution (6.5 µM in PBS) in a 96-well plate. The absorption was measured at 340 nm every 30 s for 2 h at rt (Tecan F200). All experiments were performed at least four times.

### 4.7. Cell Death and Apoptosis Induction

U251 und Mz54 glioblastoma cells (6 × 10^5^ cells/mL, 0.5 mL/well) were seeded in 24-well plates. The next day, the cells were treated with **1**, **2** or vehicle (DMSO) for 48 h. Prior to the measurement, the cells were harvested and stained using Annexin-V-APC (BD Biosciences, Heidelberg, Germany) and propidium iodide (PI, 0.05 mg/mL, Sigma-Aldrich, Taufkirchen, Germany). Cells were analyzed with BD Accuri C6 flow-cytometer (BD Biosciences), and data processing was performed using BD Accuri C6 software (BD Biosciences).

### 4.8. Immunohistochemistry

Astroglia cells grown on poly-L-lysine-coated glass cover slips were fixed with 4% paraformaldehyde in PBS for 20 min at room temperature and then permeabilized with Triton X-100 (0.1% *v*/*v* in PBS; 5 min). Nonspecific antibodies were blocked by incubation with 3% FBS in PBS (room temperature, 1 h). α-GFAP antibody (rabbit, DAKO) was used in a dilution of 1:2000 and was visualized by anti-rabbit IgG Alexa 594 (1:1000). DAPI (4′,6-diamidino-2-phenylindole) was used for nuclei staining. Fluorescence images were captured manually on a Zeiss Axiovert 200 M microscope fitted with 20- to 60-fold zoom and a Zeiss AxioCam MRm digital camera. Further applied antibodies were tubulin III (Sigma T5076), used at a dilution of 1:1000, and Annexin V. Cy3.18 (Sigma A4963).

### 4.9. Adult Organotypic Brain Slice Cultures and Ex Vivo Tumor Growth Assay

Adult organotypic brain slice culture was carried out as described previously [26,45,46]. Briefly, the brains from adult C57BL/6 J (Envigo, Horst, The Netherlands) were dissected, and dura mater was removed after the mice were euthanized. Subsequently, mouse brains were placed in warm (35–40 °C) 2% low-melting agarose (Carl Roth, Karlsruhe, Germany) and cut on a Vibratome VT1000 (Leica, Wetzlar, Germany) in 150 µm thick sections. These sections were placed on Milli-cell culture inserts (Merck KGaA, Darmstadt, Germany) and cultured in 6-well plates using FCS-free medium consisting of DMEM/F12 supplied with 1× B27 and 1× N2 supplement and 100 U/mL penicillin and 100 µg/mL streptomycin (all from Gibco, Darmstadt, Germany). One day later, multiple spheres were placed on the mouse brain slices. Adequate spheres were prepared by seeding 275.000 NCH644^GFP+^ cells in a total volume of 5 mL in a T25 flask. One day after sphere transplantation, pictures were taken (day 0), and the treatment was started, which was refreshed 3 times per week. Tumor growth was evaluated using FIJI, after which pictures were taken regularly with a Nikon SMZ25 stereomicroscope equipped with a P2-SHR Plan Apo 2 × objective operated by NIS elements software. As the tumor size was normalized to the size on day 0, growth curves were created.

## Figures and Tables

**Table 1:** Inhibitory concentrations IC_50_ (µM) ^1^ of **1** and **2** in cancer cells including several GBM cells.

Compd./Cell Line	1		2	
	24 h	48 h	24 h	48 h
518A2	0.04 ± 0.01	0.02 ± 0.01	5.0 ± 0.3	4.0 ± 0.7
HL60	0.007 ± 0.002	0.001 ± 0.000	6.8 ± 2.8	4.5 ± 1.1
KBv1^+Vbl^	0.003 ± 0.001	0.002 ± 0.000	1.6 ± 0.4	1.2 ± 0.1
MCF-7^+Topo^	0.35 ± 0.13	0.05 ± 0.01	2.5 ± 0.6	0.08 ± 0.01
HT-29	1.5 ± 0.6	0.06 ± 0.01	38 ± 4	4.0 ± 1.3
C6	1.0 ± 0.3	0.40 ± 0.06	40 ± 4	12 ± 1.3
U87	0.63 ± 0.04	0.13 ± 0.03	37 ± 3	2.7 ± 0.7
MZ-54	0.55 ± 0.16	0.06 ± 0.01	n.d.	5.48 ± 1.2
U251	0.3 ± 0.05	0.12 ± 0.02	n.d.	4.51 ± 1.3

^1^ Values are derived from dose–response curves obtained by measuring the percentage of viable cells relative to untreated controls after 24/48 h exposure of test compounds using an MTT assay. Values represent means of four independent experiments. n.d., not determined.

**Table 2:** Inhibitory concentrations IC_50_ (µM) ^1^ of **1** and **2** in non-malignant brain-derived cells.

Compd./Cell Line	1		2	
	24 h	48 h	24 h	48 h
neurons	0.60 ± 0.12	0.19 ± 0.04	26 ± 1	14 ± 2
astrocytes	1.5 ± 0.4	0.20 ± 0.03	45 ± 2	33 ± 4
NE-4C	0.40 ± 0.10	0.05 ± 0.02	7.0 ± 3.2	2.0 ± 1.2

^1^ Values are derived from dose–response curves obtained by measuring the percentage of viable cells relative to untreated controls after 24/48 h exposure of test compounds using an MTT assay. Values represent means of four independent experiments.

## Data Availability

Data supporting reported results can be obtained from the corresponding author upon request.

## Supplementary Materials

The following supporting information (stability tests) can be downloaded at: https://www.mdpi.com/article/10.3390/ijms23169056/s1.
