# Supplementary material for: Antitumor Effects of a New Retinoate of the Fungal Cytotoxin Illudin M in Brain Tumor Models
Source: Int J Mol Sci. 2022 Aug 13;23(16):9056. doi: 10.3390/ijms23169056 (PMC9408991; doi:10.3390/ijms23169056)
Supplement: Supplementary file 1 [file ijms-23-09056-s001.zip › ijms-1850487-supplementary.pdf]

Supplementary Material

Antitumor effects of a new retinoate of the fungal cytotoxin illudin M in brain tumor models

Benedikt Linder[1#], Miroslava Zoldakova[2#], Zsuzsanna Kornyei[3], Leonhard H. F. Köhler[2], Sebastian Seibt[2], Dominic Menger[1], André Wetzel[2], Emília Madarász[3], Rainer Schobert[2], Donat Kögel[1] and Bernhard Biersack[*,2]

[1]Experimental Neurosurgery, Frankfurt University Hospital, Theodor-Stern-Kai 7, 60590 Frankfurt am Main, Germany

[2]Organic Chemistry 1, University of Bayreuth, Universitaetsstrasse 30, 95440 Bayreuth, Germany

[3]Laboratory of Cellular and Developmental Neurobiology, Institute of Experimental Medicine of the Hungarian Academy of Sciences, Szigony utca 43, HU-1083 Budapest, Hungary.

Stability Tests

The stability of compound **2** in medium (DMEM) was investigated for the indicated times (Table S1). For the preparation of the calibration samples, stock solutions of the test compounds (1 mg /mL) in MeCN were initially made. The sample was diluted to 0.2 mg/mL in a mixture of MeCN/H_2O (4:1), whereof 20 µL were taken and injected into the column. For HPLC analysis, a Prontosil 120-5-C18 ace EPS 5 µm column was applied, and MeCN/H_2O + 0.1 % glacial acetic acid was used as eluent. Chromatography was run from 7030 to 9010 within 3 min for 40 min.

Table S1. Stability of compound **2** in medium.

**Sample**	**Detected Compounds (in µM)**		
	**1**	**Retinoic acid**	**2**
**2** (0 h)	<0.01	<0.01	3.92
**2** (20 h)	<0.01	<0.01	3.98
**2** (48 h)	<0.01	<0.01	3.85
**2** (144 h)	<0.01	<0.01	3.94
